# Diagnostic challenges in CADASIL

**DOI:** 10.1055/s-0043-1769618

**Published:** 2023-05-31

**Authors:** Hugh Stephen Markus

**Affiliations:** 1 University of Cambridge, Stroke Research Group, Cambridge, United Kingdom.

CADASIL (cerebral autosomal dominant arteriopathy with subcortical infarcts and leukoencephalopathy) is the most common monogenic form of stroke. It results from mutations in the *NOTCH3* gene, which encodes a transmembrane receptor. Mutations are highly stereotyped and occur in the extracellular portion of the protein, and add or remove a cysteine residue resulting in disruption of a disulphide bond in one of the epidermal growth factor like (EGF) repeats. The classical features of CADASIL include migraine usually with aura, lacunar stroke, and vascular cognitive impairment and dementia. 1 2 3 Other features include encephalopathy, epilepsy, depression and apathy.

The paper in this issue by Nogeira describes a cohort of 26 patients from Brazil with CADASIL. 4 They were recruited from 6 rehabilitation hospitals. The authors nicely describe the clinical spectrum and neuroimaging features of CADASIL. They highlight that ischaemic stroke is common, as are cognitive impairment, dementia and psychiatric manifestations. CADASIL has been described in different ethnic groups throughout the world and its clinical and radiographic appearances are broadly similar in different geographical locations, although there has been a suggestion that there may be differences in some Far Eastern pedigrees, some of whom have been reported to have non-cysteine changing mutations. 5 This study shows the pattern in Brazil is similar to that seen in other populations throughout the world.

The paper also highlights diagnostic features of CADASIL. In a patient with consistent clinical features and who often has a family history, the most useful diagnostic pointer is neuroimaging. MRI shows white matter hyperintensities which become increasingly confluent with age, and in some people lacunar infarcts and cerebral microbleeds. The pattern of WMH is characteristic with involvement of the anterior temporal pole reported in 90%. 6 This has been shown to have a high specificity. This pattern was seen in the Brazilian population. Other predilection sites include the external capsule which is commonly involved but is a less specific finding. 6 The paper also highlights diagnostic difficulties, and the fact that many cases are misdiagnosed before the correct diagnosis of CADASIL is made. Three patients in the Brazilian cohort were initially diagnosed as multiple sclerosis. The authors highlight that unlike sporadic small vessel disease, CADASIL often involves the corpus callosum which is a characteristic predilection site for multiple sclerosis. This may be one of the reasons that so many cases are misdiagnosed as multiple sclerosis. However as the authors point out oligoclonal bands in the cerebrospinal fluid are not usually present in CADASIL.

Although we have no specific treatment for CADASIL increasing evidence demonstrates that cardiovascular risk factors, particularly smoking and hypertension are associated with an increased rate of progression and earlier onset of stroke. 2 7 Therefore recent European guidelines have highlighted the need for tight cardiovascular risk factor control in this group of patients. 8

Although CADASIL is thought to be a rare disease with prevalence in the United Kingdom of 4 in 100000, 9 recent evidence suggests the typical cysteine change in mutations involving a cysteine change are much more common than in the general population. This was first shown by a group in Leiden who analysed anonymous genome sequencing databases and showed population frequencies of about 1in 1000 in Europe and even higher frequencies in the Far East. 10 Recent studies have confirmed these findings in UK Biobank and shown that these mutations are associated with an increased risk of both stroke and dementia. 11 Why typical cysteine change in mutations cause severe symptomatic familial disease in some individuals, and are apparently asymptomatic in the community in others, remains unknown. Mutation possible factors include mutation site, modifying risk factors and modifying genes. Mutations in the proximal EGF repeats have been associated with more severe disease than those in distal EGF repeats. 12 13 In UK Biobank, mutations were more likely to be associated with symptomatic stroke and dementia in those who had higher Framingham cardiovascular risk scores, 11 suggesting cardiovascular risk factors act as modifying factors, as they appear to do in patients with severe symptomatic disease. Genetic studies have suggested that modified genes also affect clinical severity, 14 and this is an area of current research interest.
